# Therapeutic potential of dihydrocapsaicin in vascular smooth muscle cell calcification: Dihydrocapsaicin curbs vascular calcification

**DOI:** 10.3724/abbs.2025143

**Published:** 2025-08-27

**Authors:** Chenxi Li, Jiajun Chen, Zhiqing Liu, Hongling Zhu, Zeyu Huang, Qingyun Zhu, Lianyong Liu, Chaobao Zhang, Xiangqi Li

**Affiliations:** 1 School of Gongli Hospital Medical Technology University of Shanghai for Science and Technology Shanghai 200093 China; 2 Traditional Chinese Medicine Department Shanghai University of Medicine & Health Sciences Shanghai 200135 China; 3 Department of Endocrinology and Metabolism Gongli Hospital Shanghai University of Medicine & Health Sciences Shanghai 200135 China; 4 Department of Endocrinology and Metabolism Punan Hospital of Shanghai Pudong New Area Shanghai 200125 China; 5 Department of Intervention Gongli Hospital Shanghai University of Medicine & Health Sciences Shanghai 200135 China; 6 Shanghai Key Laboratory of Clinical Geriatric Medicine; Department of Geriatric Medicine Huadong Hospital Shanghai Medical College Fudan University Shanghai 200040 China

Dihydrocapsaicin (DHC, C _18_H _29_NO _3_; Figure 1A) is the primary pungent component of natural capsaicinoids in chili peppers. DHC has diverse pharmacological and physiological properties, such as analgesia, anticancer, anti-inflammatory, antioxidant, and anti-obesity effects [1]. These characteristics suggest its potential clinical applications in pain relief, cancer prevention, and weight loss, with benefits for the cardiovascular and gastrointestinal systems. Consequently, investigating the pharmacological and physiological effects and mechanisms of DHC has become a research hotspot in the food and pharmacological sciences.

**Figure 1:**
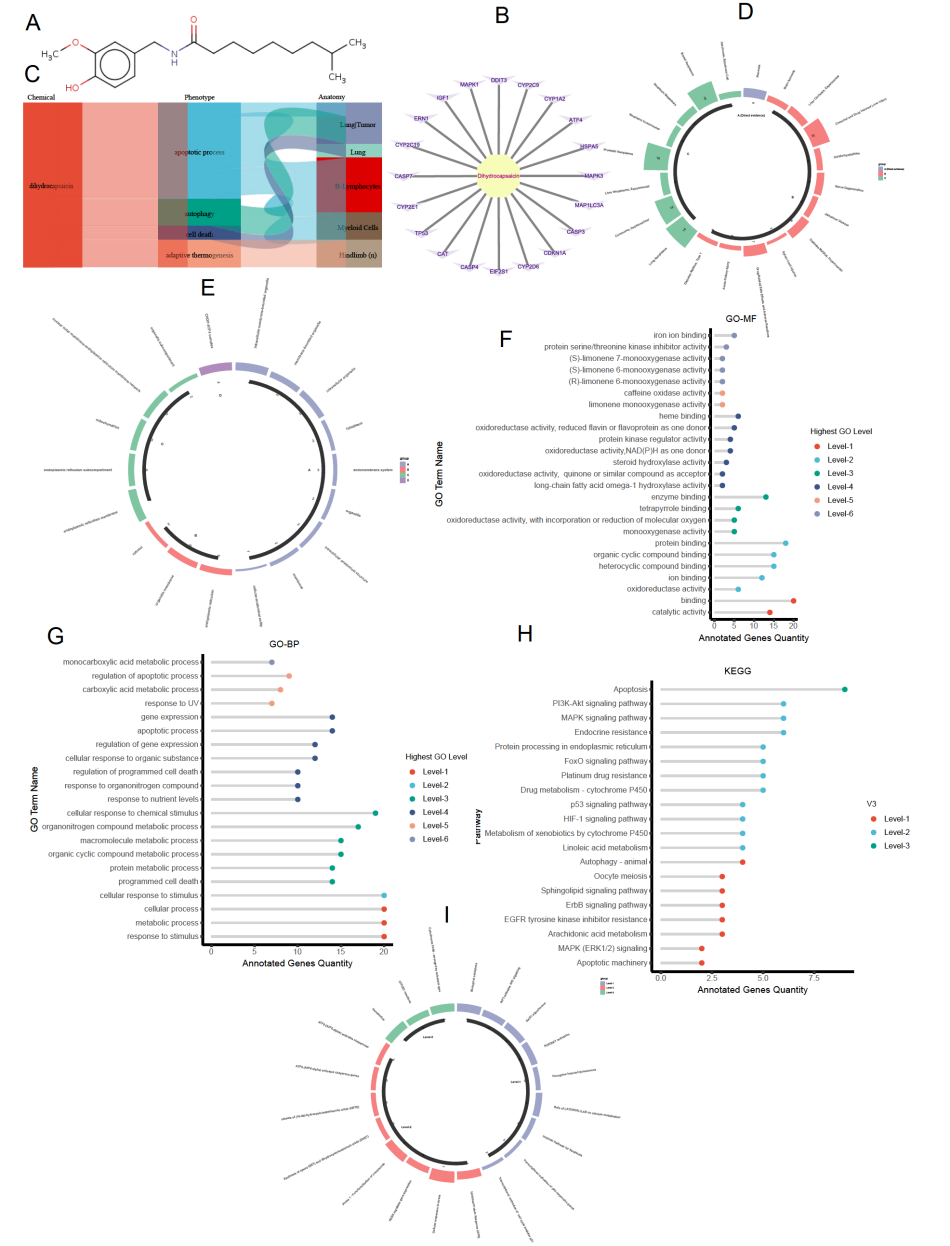
Figure 1 Analysis of the functions and pathways of DHC-targeting genes (A) Molecular structure of DHC from BindingDB. (B) Targeting genes of DHC. (C) Chemical-Phenotype analysis for DHC. (D) Disease analysis for DHC-targeting genes. (E) CC analysis of GO for DHC-targeting genes. (F) MF analysis of GO for DHC-targeting genes. (G) BP analysis of GO for DHC-targeting genes. (H) KEGG pathway analysis for DHC-targeting genes. (I) REACTOME analysis for DHC-targeting genes. CC: Cellular Compartment; MF: Molecular Function; BP: Biological Process; GO: Gene Ontology; KEGG: Kyoto Encyclopedia of Genes and Genomes; DHC: Dihydrocapsaicin.

Vascular calcification (VC) involves the accumulation and deposition of hydroxyapatite crystals within arterial wall cells, resulting in increased vascular wall stiffness, decreased compliance, thrombosis, and plaque rupture. VC is a common adverse phenotype of various vascular lesions; is a phenotypic feature of diseases such as atherosclerosis, hypertension, aortic valve stenosis, coronary artery disease, diabetes mellitus, and chronic kidney disease; and is an independent risk factor for disease occurrence, progression, and mortality [2]. Moreover, VC is also an independent risk factor and a sign of disease exacerbation and aging. Therefore, it is a life-threatening driver of human health. However, there is currently no acknowledged effective therapeutic strategy to reverse or cure VC clinically, particularly when a specific drug is lacking [3]. Thus, identifying a drug that inhibits VC holds significant importance and clinical value.

Given the various pharmacological effects of DHC, we hypothesized a potential connection between DHC and VC. To investigate the roles of DHC and its related pathways in VC, we utilized the Comparative Toxicogenomics Database (CTD) and its online tools to extract experimental (not computer-predicted) target genes of DHC. Specifically, we utilized the ‘Search’ window in the Comparative Toxicogenomics Database (CTD) to obtain comprehensive data on dihydrocapsaicin (DHC) using default search parameters. The retrieved data were subsequently processed and visualized using SangerBox, HIPLOT and Cytoscape tools. We further analyzed its cellular functions and signaling pathways using Gene Ontology (GO), Kyoto Encyclopedia of Genes and Genomes (KEGG) and REACTOME analyses.

Our results demonstrated that DHC has 20 target genes, including *ATF4* , *CASP3*, *CASP4*, *CASP7*, *CAT*, *CDKN1A*, *CYP1A2*, C *YP2C19* , *CYP2C9*, *CYP2D6*, *CYP2E1*, *DDIT3*, *EIF2S1*, *ERN1*, *HSPA5* , *IGF1*, *MAP1LC3A*, *MAPK1*, *MAPK3*, and *TP53* ( Figure 1B). Chemical-phenotype analysis of DHC revealed cell death phenotypes, such as apoptotic processes and autophagy ( Figure 1C). Disease analysis revealed three types: necrosis, tumors, and common diseases such as cardiomyopathies ( Figure 1D). Cellular component analysis revealed that these target genes are located primarily in the cellular membrane system. The genes associated with the highest GO levels include the CHO-ATF4 complex, endoplasmic reticulum membrane, and intracellular membrane-bound organelles ( Figure 1E). Molecular function analysis highlighted energy and substance metabolism, stress responses, antioxidant mechanisms, enzyme activity, and signal transduction ( Figure 1F). Biological process analysis revealed processes related to metabolism, apoptosis, and responses to various stimuli ( Figure 1G). KEGG analysis revealed pathways involved in metabolism, cell death, and critical signaling pathways, such as the MAPK, ErbB, HIF-1, FoxO, and sphingolipid signaling pathways ( Figure 1H). REACTOME analysis corroborated the results of the GO and KEGG analyses ( Figure 1I). These findings suggest that the roles and signaling pathways of DHC are highly relevant to VC, prompting us to experimentally verify the modulatory effect of DHC on VC.

Then, we experimentally validated the inhibitory effect of DHC on VC using a simple VC cellular model we previously established [4]. Briefly, human vascular smooth muscle cells (hVSMC; NEWGAINBI, Shanghai, China) were cultured in 24-well plates with α-MEM complete medium (Thermo Fisher Scientific, Waltham, USA). Upon reaching 80%–90% confluence, the cells were induced to calcify by adding α-MEM basal medium + CaCl _2_ (by supplementation with 1.2 μL of 100 mM CaCl _2_ solution), combined with different concentrations of DHC (by supplementation with 0 μL, 0.5 μL, 2 μL, or 8 μL of 4 mM DHC solution; Aladdin, Shanghai, China) as a calcification modulator, to the experimental group in a total volume of 400 μL, while cells treated with α-MEM complete medium + H _2_O were used in the control group. After 3–6 h of induction, the medium was removed, and cells were washed twice with 1× TBS, fixed with 4% neutral formaldehyde for 30 min, and washed again with 1× TBS, followed by staining with Alizarin Red (pH 8.0; Cyagen, Suzhou, China) for 5 min. Finally, cells were washed three more times with 1× TBS and observed under a TS100 microscope (Nikon, Tokyo, Japan), with images captured via a CCD camera ( Figure 2A–E). The quantitative evaluation was performed using ImageJ software (version V1.8.0.112), and the results are shown in Figure 2F, confirming the significant inhibitory effect of DHC on VC.

**Figure 2:**
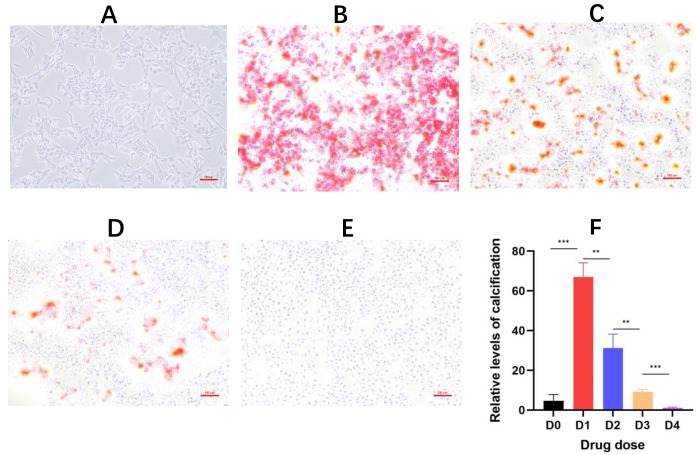
Figure 2 DHC curbs cellular calcification (A) Normal culture of hVSMCs with basal medium. (B–E) Addition of DHC at different concentrations. (F) Quantitative comparison of cellular calcification levels for (A–E). Concentrations used: D0: not induction; D1: 0 μM; D2: 5 μM; D3: 20 μM; D4: 80 μM. n = 3; **P < 0.01, *** P < 0.001. After 4.5 h of induction and drug administration, alizarin red staining was performed. DHC: dihydrocapsaicin; hVSMCs: human vascular smooth muscle cells; BM: basal medium.

Recent evidence has shown that VC is not a passive irreversible comorbidity but an active process regulated by many factors [3]. This provides a good chance to actively prevent or treat VC. Programmed cell death, such as apoptosis, is a key promoter of calcification. The CHOP-ATF4 complex is a critical regulator of osteoblast differentiation, and the endoplasmic reticulum serves as a Ca ^2+^ reservoir, both of which are closely related to calcium signaling. Various stresses, such as ischemia, exogenous toxins, and disorders of calcium and phosphorus metabolism, can lead to cellular calcification. Our analysis revealed that the roles and signaling pathways of DHC-targeting genes are highly related to metabolism, cell death, and responses to various stimuli, suggesting that there might be a strong association between DHC and cellular calcification. These findings are consistent with the conclusions concerning the roles of inflammatory responses, endoplasmic reticulum stress, mitochondrial dysfunction, iron homeostasis, metabolic imbalance, and some related signaling pathways in VC progression [3]. The critical point is that the DHC receptor, TRPV family (TRPV1–6), constitutes a time- and Ca ^2^-dependent non-selective cation channel linked to the TRP ion channel family [5]. This finding implies that DHC can inhibit the influx of Ca²⁺ from outside the cell. Our model, which uses high calcium without serum, effectively simulates ischemia, exogenous toxins, and calcium metabolism disorders, enabling rapid induction of cellular calcification within hours [4]. This model is especially well suited for investigating vascular calcification associated with acute ischemic events, such as myocardial infarction or stroke, with a focus on contributing factors such as a sedentary lifestyle and reduced physical activity. This model surpasses traditional cell calcification models that are complex and unstable, taking up to one month. Therefore, it is highly suitable for screening drugs that inhibit calcification. To our knowledge, DHC is the first model-confirmed natural product from an edible plant, the chili pepper, to emerge as a potential calcification-inhibiting drug. Future animal and clinical experiments should be conducted to confirm the cellular results.

In summary, our study elucidated the roles and signaling pathways of DHC in cellular calcification. By employing various analytical tools and methods, we determined that the roles and signaling pathways of DHC are predominantly focused on cell death, metabolism, and responses to various stresses, all of which are highly relevant to the calcification process. Cellular experiments confirmed its inhibitory effect on VC. Our research not only provides the first potential model-confirmed natural food-level drug from chili peppers for treating calcification-related diseases but also exemplifies a rapid strategy for screening drugs for various diseases associated with calcium metabolism disorders.

## Supporting information

25497SUPPLE_METHOD

## Supplementary Data

Supplementary data is available at *Acta Biochimica et Biophysica Sinica* online.
