# Supplementary material for: Therapeutic potential of dihydrocapsaicin in vascular smooth muscle cell calcification: Dihydrocapsaicin curbs vascular calcification
Source: Acta Biochim Biophys Sin (Shanghai). 2025 Aug 27;58(4):925–8. doi: 10.3724/abbs.2025143 (PMC13107027; doi:10.3724/abbs.2025143)
Supplement: 25497SUPPLE_METHOD [file 25497SUPPLE_METHOD.docx]

**Supplementary methods**

Human vascular smooth muscle cells were cultured in 24-well plates with α-MEM complete medium. Upon reaching 80%−90% confluence, the cells were induced to calcify by adding α-MEM basal medium + CaCl_2_ (100 mM) to the experimental group and α-MEM complete medium + H_2_O to the control group. After 3−6 h of induction, the medium was removed, and cells were washed twice with 1× TBS, then fixed with 4% neutral formaldehyde for 30 min. Subsequent washing with 1× TBS was followed by 5-min Alizarin Red staining. Finally, cells were washed three more times with 1× TBS and observed under a Nikon TS100 microscope (Tokyo, Japan), with images captured via a CCD camera. Alizarin Red staining of calcium nodules indicated cell calcification.
